# Supplementary material for: The challenges of containing SARS-CoV-2 via test-trace-and-isolate
Source: Nat Commun. 2021 Jan 15;12:378. doi: 10.1038/s41467-020-20699-8 (PMC7810722; doi:10.1038/s41467-020-20699-8)
Supplement: Supplementary file 1 — Supplementary Information [file 41467_2020_20699_MOESM1_ESM.pdf]

The challenges of containing SARS-CoV-2 via test-trace-and-isolate (Supplementary Information)

Sebastian Contreras[1, 2, X], Jonas Dehning[1, X], Matthias Loidolt[1, X], Johannes Zierenberg[1], F. Paul Spitzner[1], Jorge H. Urrea-Quintero[1], Sebastian B. Mohr[1], Michael Wilczek[1,3], Michael Wibral[4], and Viola Priesemann[1,3*]

[1]Max Planck Institute for Dynamics and Self-Organization, Am Faßberg 17, 37077 Göttingen, Germany.
[2]Centre for Biotechnology and Bioengineering, Universidad de Chile, Beauchef 851, 8370456 Santiago, Chile.
[3]Institute for the Dynamics of Complex Systems, University of Göttingen, Friedrich-Hund-Platz 1, 37077 Göttingen, Germany.
[4]Campus Institute for Dynamics of Biological Networks, University of Göttingen, Hermann-Rein-Straße 3, 37075 Göttingen, Germany.
[X]These authors contributed equally: Sebastian Contreras, Jonas Dehning, Matthias Loidolt

Supplementary Note 1: Linear stability analysis

For analyzing the stability of the governing differential equations, namely, whether an outbreak could be controlled, we studied the linear stability of the system. The linearized system for equations (1)-(3) of the main manuscript, with limitless tracing capacity, is given by:

$$\frac{d}{dt}\begin{pmatrix} T \\ H \\ H^s \end{pmatrix} = \begin{pmatrix} \Gamma\left(\nu R_t^H - 1\right) & \lambda_r\left(\eta R_t^H + 1\right) & \lambda_s\left(1 + \eta R_t^H\right) \\ \Gamma\epsilon R_t^H & \Gamma\left(R_t^H - 1\right) - \lambda_r\left(1 + \eta R_t^H\right) & -\lambda_s\left(1 + \eta R_t^H\right) \\ (1-\xi^{\mathrm{ap}})\,\Gamma\epsilon R_t^H & (1-\xi^{\mathrm{ap}})\left(\Gamma R_t^H - \lambda_r\left(1 + \eta R_t^H\right)\right) & -\eta\,(1-\xi^{\mathrm{ap}})\,R_t^H\lambda_s - (\lambda_s + \lambda_r + \Gamma) \end{pmatrix}\begin{pmatrix} T \\ H \\ H^s \end{pmatrix} \tag{1}$$

By studying the eigenvalues of the associated matrix, we can infer the stability of the solutions around the equilibrium. In particular, we define R_{crit}^H as the largest R_t^H such that the real part of $\mu_{\max}$, the largest eigenvalue of matrix A, is strictly negative.

Supplementary Note 2: Equilibrium equations for case numbers below tracing capacity

A system equilibrium is reached when time derivatives equal to zero. That is by setting the left-hand side in eqs. (1)-(3) of the main manuscript equal to zero, e.g., $dT/dt = 0$. Regarding the SIR-like model presented here, an equilibrium with non-zero new cases can be attained for a constant, positive, influx Φ and a particular combination of the parameters, and depending on whether the health authority's tracing capacity is exceeded or not. This equilibrium exhibits a stable number of daily new cases $\hat{N}^{\mathrm{obs}} = N_\infty^{\mathrm{obs}}$, which, excluding random testing ($\lambda_r = 0$), would take the form:

$$N_\infty^{\mathrm{obs}} = \Gamma\nu R_t^H T_\infty + \lambda_s H_\infty^s + f(H^s, H), \tag{2}$$

where $f(H^s, H)$ is defined by equation (5) in the main manuscript. The convolution of reporting delays would not play a significant role (as cases would be constant).

*viola.priesemann@ds.mpg.de

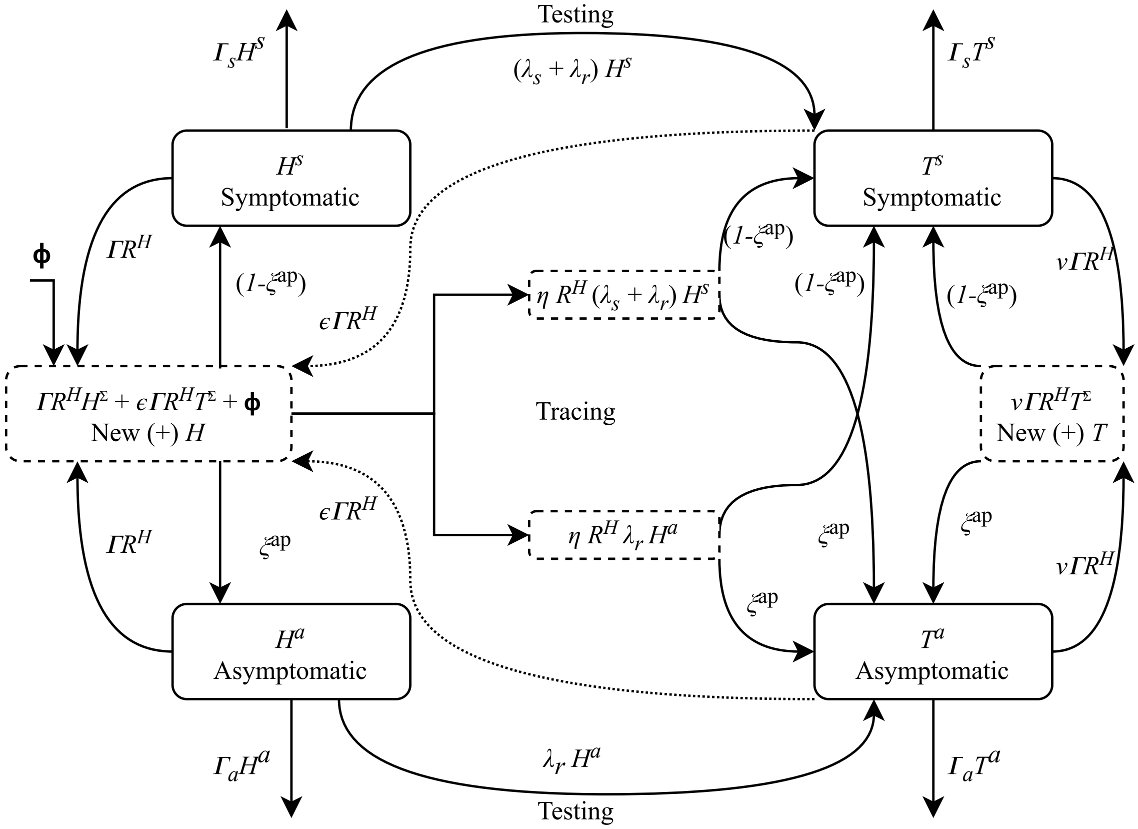

Supplementary Figure 1: **Flowchart of the complete model.** This figure corresponds to Fig. 1 in the main manuscript.

For the case in which the tracing capacity is not exceeded ($\eta\lambda_s R_t^H H_\infty^s < n_{\max}$), and excluding random testing ($\lambda_r = 0$), setting equations eqs. (1)-(3) of the main manuscript equal to zero and expanding equation (2) gives the following set of equations:

$$T_\infty = \frac{\lambda_s\left(1+\eta R_t^H\right)}{\Gamma\left(1-\nu R_t^H\right)} H_\infty^s \tag{3}$$

$$H_\infty = H_\infty^s \frac{\lambda_s}{\Gamma}\left(\frac{\frac{\Gamma}{\lambda_s}+\xi^{\text{ap}}}{1-\xi^{\text{ap}}}\right) \tag{4}$$

$$H_\infty^s = \frac{\Phi}{\lambda_s\left(1+\eta R_t^H\right)}\left[\left(\frac{\epsilon R_t^H}{\nu R_t^H-1}+R_t^H\right)-\left(R_t^H-1\right)\frac{\eta R_t^H+\frac{1+\Gamma/\lambda_s}{1-\xi^{\text{ap}}}}{\eta R_t^H+1}\right]^{-1} \tag{5}$$

$$N_\infty^{\text{obs}} = \Gamma\nu R_t^H T_\infty + \lambda_s H_\infty^s\left(1+\eta R_t^H\right). \tag{6}$$

To calculate N_∞^{obs} in terms of the model's parameters, we insert equations (3)– (5) into equation (6):

$$N_\infty^{\text{obs}} = \lambda_s H_\infty^s\left(1+\eta R_t^H\right)\left(\frac{1}{1-\nu R_t^H}\cancel{-1+1}\right), \tag{7}$$

$$= \lambda_s H_\infty^s \frac{1+\eta R_t^H}{1-\nu R_t^H}. \tag{8}$$

This equilibrium is stable as soon as $R_t^H < R_{\text{crit}}^H$ (R_{crit}^H is presented in Table 1 of the main manuscript).

To study the effect of Φ on the steady-state observed case numbers N_∞^{obs}, we evaluate the value of H_∞^s in equation 8 using equation 5:

$$\frac{\Phi\lambda_s \cancel{\left(1+\eta R_t^H\right)}}{\left(1-\nu R_t^H\right)\lambda_s \cancel{\left(1+\eta R_t^H\right)}} \left[\left(\frac{\epsilon R_t^H}{\nu R_t^H - 1} + R_t^H\right) - \left(R_t^H - 1\right) \frac{\eta R_t^H + \frac{1+\Gamma/\lambda_s}{1-\xi^{\text{ap}}}}{\eta R_t^H + 1}\right]^{-1} = N_\infty^{\text{obs}}, \tag{9}$$

concluding that they are in direct proportionality, with a constant of proportionality k depending on the system's parameters:

$$\frac{N_\infty^{\text{obs}}}{\Phi} = k\left(\xi^{\text{ap}}, \lambda_s, \eta, \Gamma, \nu, \epsilon, R_t^H\right). \tag{10}$$

Equilibrium equations for case numbers above tracing capacity

We can also derive equilibrium equations for the case where tracing capacity is exceeded ($\eta\lambda_s R_t^H H_\infty^s > n_{\text{max}}$). Remark that in this case, that critical reproduction number at which the equilibrium is stable is smaller than for the $\eta\lambda_s R_t^H H_\infty^s < n_{\text{max}}$ case.

When the tracing capacity is exceeded, the values returned by function $f(H^s, H)$ (defined by equation 5 in the main manuscript) are constant $f(H^s, H) = n_{\text{max}}$. Then, setting the equations (1)-(3) of the main manuscript equal to zero leads to:

$$T_\infty = \frac{\lambda_s H_\infty^s + n_{\text{max}}}{\Gamma\left(1-\nu R_t^H\right)} \tag{11}$$

$$H_\infty = H_\infty^s \frac{\lambda_s}{\Gamma}\left(\frac{\frac{\Gamma}{\lambda_s} + \xi^{\text{ap}}}{1-\xi^{\text{ap}}}\right) \tag{12}$$

$$\lambda_s H_\infty^s = \frac{n_{\text{max}}\left(\frac{\epsilon R_t^H}{\nu R_t^H - 1} + 1\right) - \Phi}{\left(R_t^H - 1\right)\left(\frac{\frac{\Gamma}{\lambda_s} + \xi^{\text{ap}}}{1-\xi^{\text{ap}}}\right) - \left(1 - \frac{\epsilon R_t^H}{1-\nu R_t^H}\right)} \tag{13}$$

Similarly, we can derive an equation for N_{max}, which represents the **maximum observed number of cases** at the tracing capacity limit, by using the new equilibrium values and the tracing-limit condition $f(H^s, H) = n_{\text{max}}$ in equation (2):

$$N_\infty^{\text{obs}} = \frac{\left(R_t^H - 1\right)\left(\frac{\frac{\Gamma}{\lambda_s} + \xi^{\text{ap}}}{1-\xi^{\text{ap}}}\right) n_{\text{max}} - \Phi}{\left(R_t^H - 1\right)\left(\frac{\frac{\Gamma}{\lambda_s} + \xi^{\text{ap}}}{1-\xi^{\text{ap}}}\right) - \left(1 - \frac{\epsilon R_t^H}{1-\nu R_t^H}\right)} \frac{1}{1-\nu R_t^H} \overset{!}{=} N_{\text{max}}. \tag{14}$$

Note that this approach to calculate N_{max} assumes the system is stable and has a finite equilibrium value. When the system is out of equilibrium, the value N_{max} is only an approximation for the number of observed cases at which tracing capacity is overwhelmed.

Supplementary Note 3: Parameter uncertainty propagation

Supplementary Table 1: Parameter uncertainty propagation

Parameter	Meaning	Mean	95% CI	α	β	Dist.	Units
ξ^{ap}	Apparent Asymptomatic ratio	0.32	0.19–0.47	13.1	27.8	beta	–
λ_s	Symptom-driven test rate	0.10	0.05–0.16	10.7	96.3	beta	days^{-1}
ν	Isolation factor (traced)	0.10	0.03–0.22	3.5	31.5	beta	–
η	Tracing efficiency	0.66	0.59–0.73	117.9	60.7	beta	–
ϵ	Missed contacts (traced)	0.10	0.03–0.22	3.5	31.5	beta	–
$R^H_{\mathrm{crit}}\big\|_{\eta=0.66}$	Critical reproduction number (hidden) (with $<\eta>= 0.66$)	1.90	1.42–2.70	–	–	–	–
$R^H_{\mathrm{crit}}\big\|_{\eta=0}$	Critical reproduction number (hidden) (with $\eta = 0$)	1.42	1.23–1.69	–	–	–	–

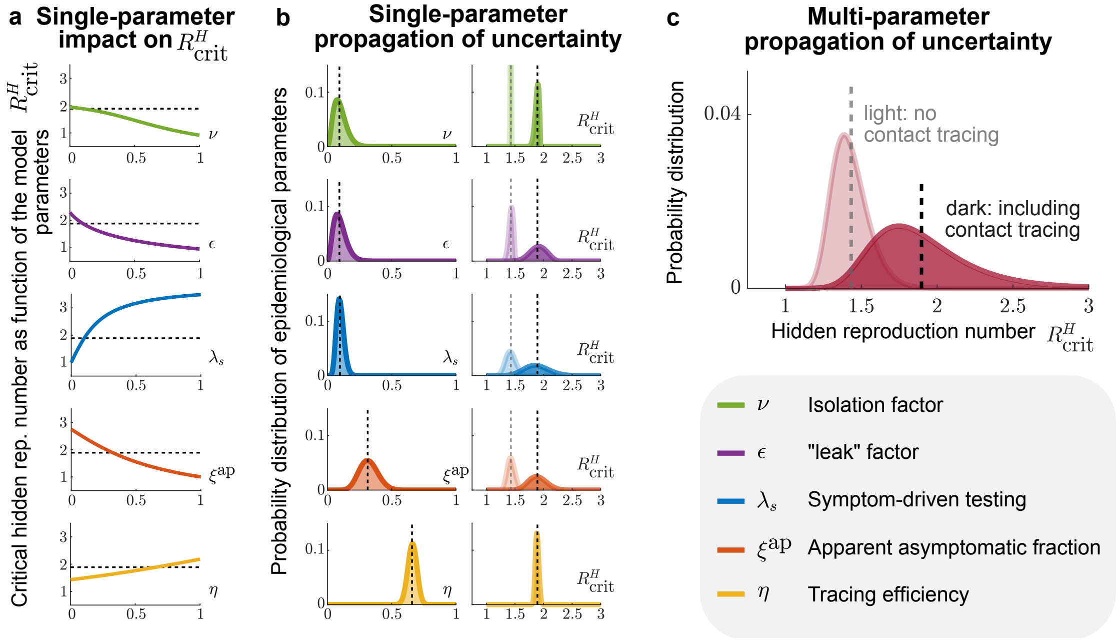

Supplementary Figure 2: **Propagation of TTI-parameter uncertainties to the critical reproduction number.** As the different parameters involved in our model play different roles, the way their variability propagates to R^H_{crit} differs, even when their variability profiles look similar. **(a)** Impact of single-parameter variation on the critical hidden reproduction number R^H_{crit}. To evaluate the monotony (direction) of their impact on R^H_{crit}, we scan their entire definition range, ignoring the practical feasibility of achieving such values. The dotted black line shows the default critical hidden reproduction number. **(b)** Univariate uncertainties of TTI parameters modeled by beta distributions centered on their default value (dotted black line), and the resulting distribution of critical reproduction numbers R^H_{crit} (right column). Results are shown assuming testing only (light colors) or testing and tracing (dark colors). The default value of R^H_{crit} is marked by the dotted lines in the presence (black) or absence (gray) of tracing. **(c)** Distribution of critical reproduction numbers arising from multivariate uncertainty propagation given by the joint of the distributions shown in (a) for testing only (light colors), or testing and tracing (dark colors). The default value of R^H_{crit} is marked by the dotted lines in the presence (black) or absence (gray) of tracing. Results show averages of 100 000 realizations.

Supplementary Note 4: Studying the effect of different influx scenarios on the TTI-conditional stability of the system

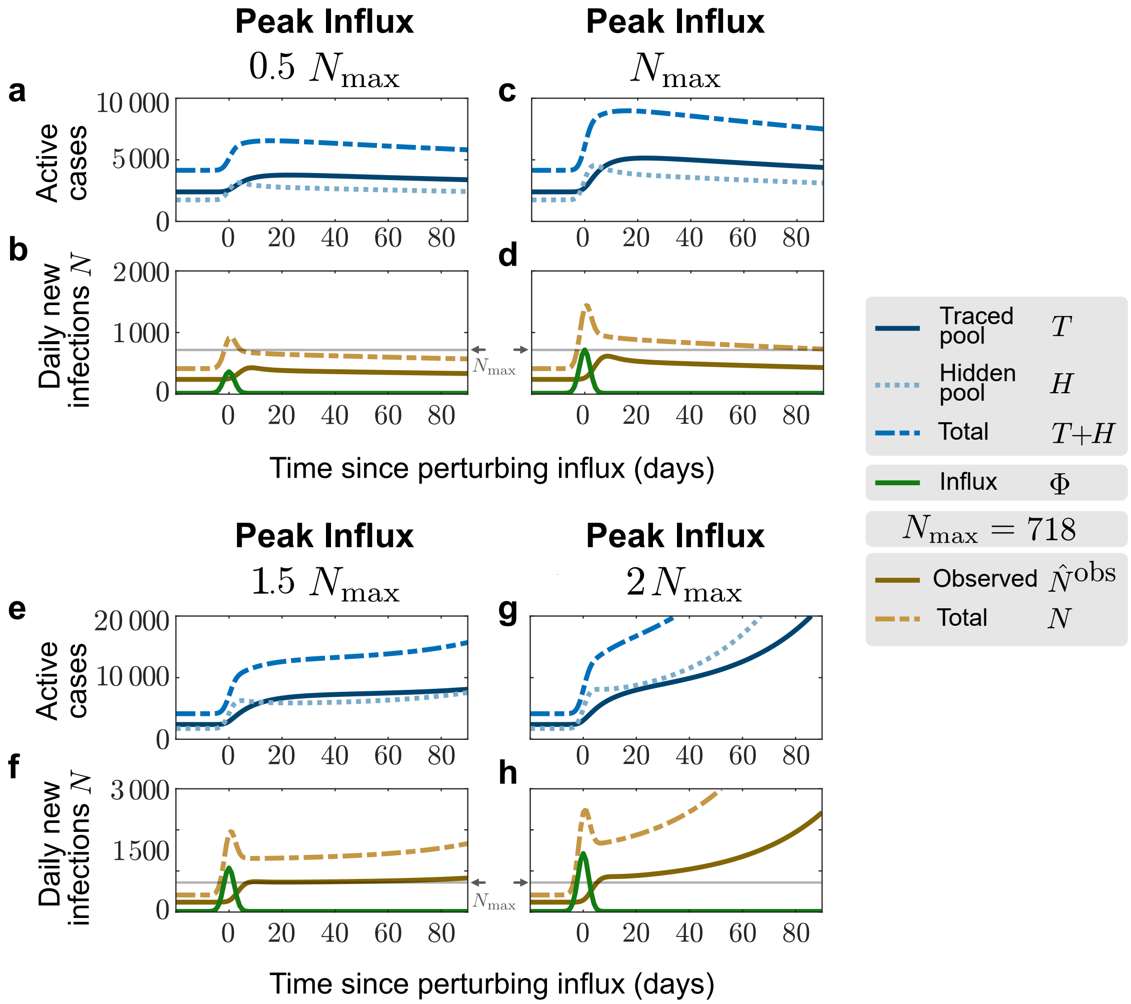

Supplementary Figure 3: **Effect of influx amplitude on the stability of the system.** This figure corresponds to Fig. 3 in the main text. For the default capacity scenario, we explore influxes of different amplitude: the peak of the influx is equal to 0.5 **(a,b)**, 1.0 **(c,d)**, 1.5 **(e,f)**, and 2 **(g,h)** times the -equilibrium- capacity limit of the health authorities $N_{\max}$. The influx is normally spread around day 0 with standard deviation $\sigma = 2$ days, corresponding to the 92 % of a total influx of, respectively, 1773, 3546, 5319, and 7092 individuals, entering the system over 7 days.

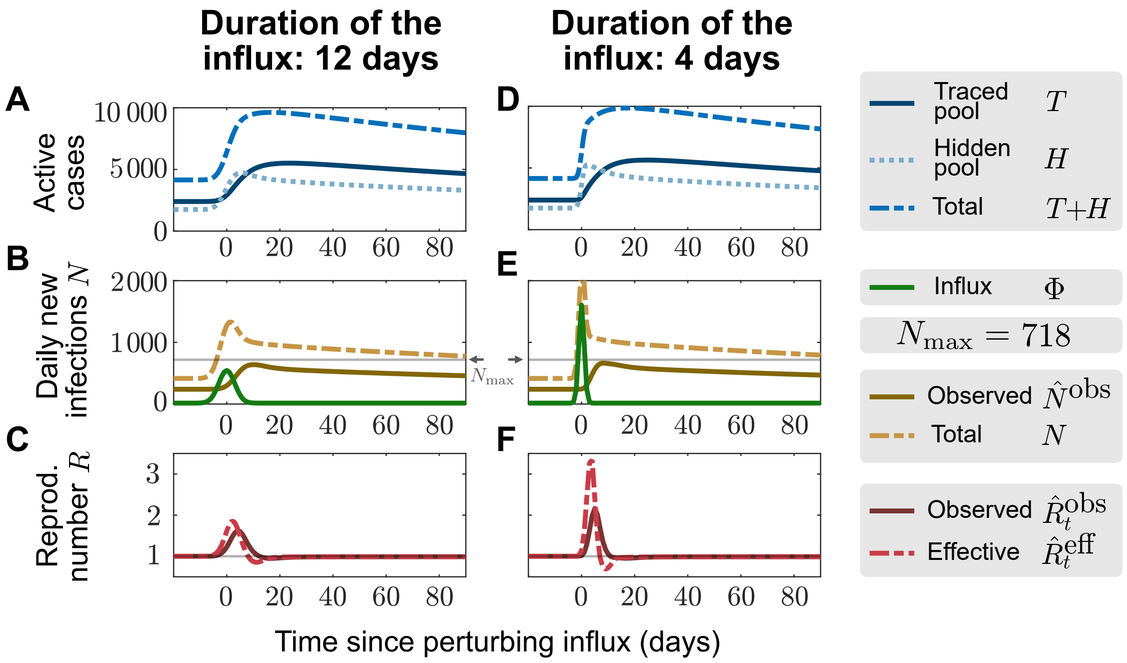

Supplementary Figure 4: **Effect of influx duration on the stability of the system.** This figure corresponds to Fig. 3 in the main text. For the default capacity scenario, we explore influxes of identical overall number spread over different time windows: 92 % of the 4000 infections enter the system in 12 days ($\sigma = 3$ days, **a-c**); or in 4 days ($\sigma = 1$ day,**d-f**).

Supplementary Note 5: Testing and tracing give rise to two stabilized regimes of spreading dynamics

The simple SIR model with external influx exhibits two regimes: stable or growing: If the reproduction number R is less than one (blue region in Supplementary Fig. 5a.b), each new case infects less than one new case on average, and the number of new cases in equilibrium $\hat{N}^{\text{obs}}_\infty$ is finite (solid blue line in Supplementary Fig. 5a). If R is above one (red region in Supplementary Fig. 5a,b), each new case infects more than one new case, and the number of new cases grows quickly. These regimes are reflected in the equilibrium observed reproduction number $\hat{R}^{\text{obs}}_\infty$: In the stable regime, $\hat{R}^{\text{obs}}_\infty = 1$, and in the unstable regime $\hat{R}^{\text{obs}}_\infty > 1$ (solid black line in Supplementary Fig. 5b).

Distinct from the standard SIR model, our two-pool model with TTI exhibits two TTI-stabilized regimes of spreading dynamics: The first regime requires only to isolate persons with positive test results ("testing-stabilized"), the second requires, in addition, to find and isolate contacts of a positively tested person ("tracing-stabilized", Supplementary Fig. 5c,d). Due to the stabilization, the transition to instability for these two regimes is shifted towards hidden reproduction numbers R_t^H above one (dotted grey lines in Supplementary Fig. 5c). As in the classical stable regime, the number of new cases in equilibrium $\hat{N}^{\text{obs}}_\infty$ diverges when approaching these critical points (dashed green and dotted orange lines in Supplementary Fig. 5c). The ultimately unstable regime begins at $R_t^H = R_{\text{crit}}^H \simeq 1.9$ for our default parameters. Note that R_{crit}^H is below the basic reproduction number reported for SARS-CoV-2 ($R_0 \approx 3.3$ [1–3]), however, it may already be attained by reducing contacts by 40 % from levels at the beginning of the pandemic.

Supplementary Note 6: A limited tracing capacity renders the tracing-stabilized regime meta-stable.

The amount of contacts that can reliably be traced by health authorities is limited due to the work to be performed by trained personnel: Contact persons have to be identified, informed, and ideally also counseled during the preventive quarantine. Exceeding the tracing capacity limit destabilizes an otherwise stable regime, rendering it effectively meta-stable (amber in Supplementary Fig. 5c,d. Once the tracing capacity is exceeded,

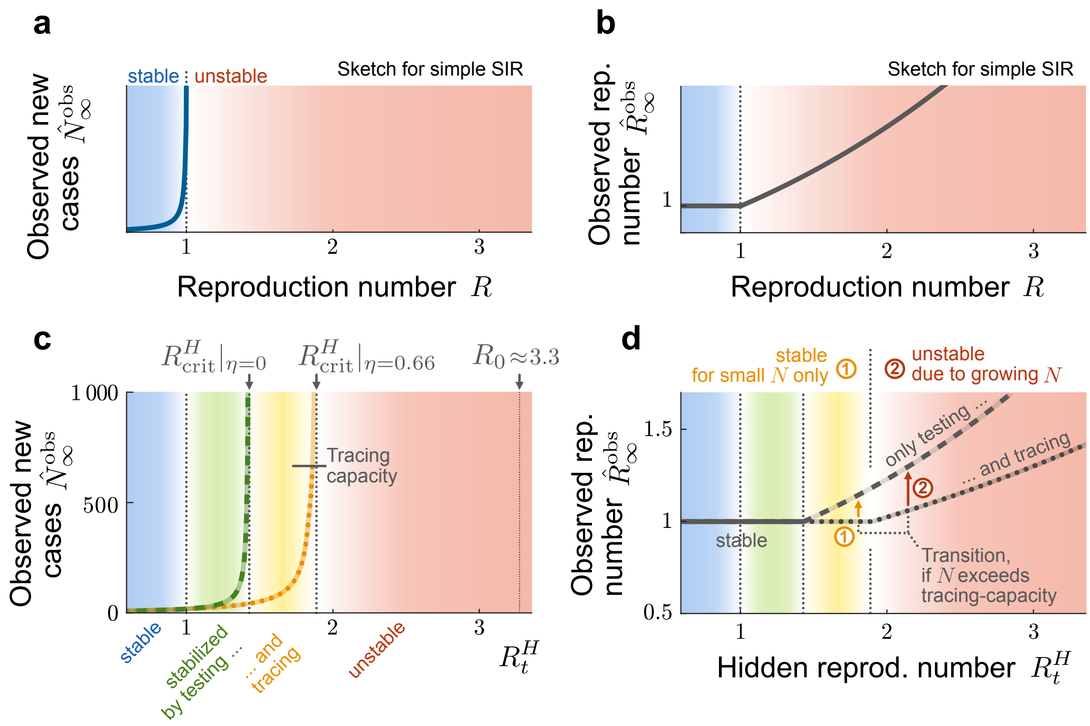

Supplementary Figure 5: **Testing and tracing give rise to two TTI-stabilized regimes of spreading dynamics.** This figure is a more detailed version of Fig. 5 in the main text. In the simple SIR model with external influx **(a,b)**, the spreading dynamics exhibit a stable and unstable regime (blue and red regions, respectively). In addition to these, our two-pool model exhibits **(c,d)** two "TTI-stabilized" regimes that arise from the isolation of infected persons upon testing positive (green region) or upon being traced as a contact of an infected person (amber region). **(a)** Observed case numbers $\hat{N}_\infty^{\text{obs}}$ in the simple SIR model with external influx approach a finite equilibrium in the stable regime (solid blue line). As the reproduction number R approaches the critical point at $R = 1$, the case numbers in equilibrium $\hat{N}_\infty^{\text{obs}}$ diverge, growing uncontrolled in the unstable regime. **(b)** The asymptotic observed reproduction number $\hat{R}_\infty^{\text{obs}}$ inferred from the observed new cases $\hat{N}_\infty^{\text{obs}}$ in the simple SIR model with external influx is always 1 in the stable regime. However, it reflects the real value of R in the unstable regime (solid grey line). **(c)** Daily number of new infections $\hat{N}_\infty^{\text{obs}}$ in our two-pool model are finite in the stable and stabilized regimes, but diverge upon approaching the critical points of the "testing only" or "testing and tracing" strategies (dashed green and dotted orange lines, respectively). They are infinite in the unstable regime or when the tracing capacity limit is reached (black bar). The exact position of the critical points of the stabilized regimes depend on the efficiencies of the respective strategies: Symptom-driven testing alone ($\eta = 0$, green) can only stabilize the spread for $\hat{R}_\infty^{\text{obs}} < R^H_{\text{crit}}|_{\eta=0} \approx 1.5$, while symptom-driven testing and tracing ($\eta = 0.66$, amber) can stabilize the spread for up to $R_t^H < R^H_{\text{crit}}|_{\eta=0.66} \approx 1.9$ for our default parameters (Table. 1 in the main text). **(d)** The observed reproduction number $\hat{R}_\infty^{\text{obs}}$ of a system stabilized by symptom-driven testing and tracing is always 1 in the "stable" and "testing-stabilized" regimes (solid grey line). In the meta-stable "testing-and-tracing-stabilized" regime (dotted grey line), $\hat{R}_\infty^{\text{obs}} = 1$ as long as the tracing capacity is not exceeded. If exceeded, the system behaves asymptotically as if there was only symptom-driven testing in place (transition ①, see also Fig. 3), which can only slow down, but not control the spread anymore. In the "unstable" regime, the observed reproduction number $\hat{R}_\infty^{\text{obs}}$ always increases with R_t^H – thus, the number of cases always grows. As long as the tracing capacity is not exceeded by this growth, testing-and-tracing slows down the spread (dotted grey line) – afterward, the system behaves asymptotically as if there was only symptom-driven testing slowing down the spread (transition ②, see also Fig. 6). The curves showing observed new cases are obtained from the analytical description of the equilibrium for unlimited tracing capacity (equations (3) - (5)). The curves showing the observed reproduction number are obtained from the linear stability analysis (equation (1)).

the system will behave asymptotically as if it had testing only, i.e. the effective and observed reproduction number will strongly increase (transition ①) from dotted to dashed grey line in Supplementary Fig. 5d).

This demonstrates that a low number of new infections is essential to control the spread when $R_t^H > 1$. Crossing the capacity limit of tracing, $N_{\max}$, leads to a self-accelerating spread, and thereby presents

a qualitatively new tipping point to instability in an otherwise stable system. - rendering it effectively meta-stable.

The transition from the metastable regime to the unstable regime happens when the tracing system is overwhelmed due to the number of observed new cases exceeding the tracing capacity ($\hat{N}^{\text{obs}} > N_{\text{max}}$). This can occur because of an increased influx Φ of infected people, e.g. returning from holiday or a super-spreading event.

Supplementary Note 7: Incorporating different transmissibility of asymptomatic and symptomatic cases.

In the main text, we assumed that hidden asymptomatic and symptomatic infections would spread with identical reproduction number R_t^H. In reality, asymptomatic cases tend to have a lower viral load but might have more contacts than hidden symptomatic cases because they do not decrease their movement after falling sick. Consequently, asymptomatic infections could spread with lower or higher reproduction numbers than symptomatic cases.

We can model this difference in reproduction number by introducing an effective relative transmissibility factor χ: $R_t^{H,a} = \chi R_t^{H,s}$. The exact value of this factor is a function of the viral load, the infection time, and the number of contacts, which we all do not model explicitly. Additionally, our model treats “real” and “apparent” asymptomatic cases (those that do not get tested) in the same manner, which should also be noted when interpreting the exact value of χ. Incorporating all of these influences into a single effective factor allows us to study how the relative transmissibility of “apparent” asymptomatic cases impacts the stability of the system.

Regardless of the relative transmissibility of asymptomatic and symptomatic cases, the overall reproduction number R_t^H can be inferred from the average spread in the population. Therefore, it must stay the same for all values of χ, which can be achieved by rescaling the transmissibility of symptomatic cases appropriately. The correct rescaling can be found from the following equation, which relates the reproduction numbers in an equilibrium state where asymptomatic carriers occupy a fraction ξ^{ap} of the total infections:

$$R_t^H = R_t^{H,a} \xi^{\text{ap}} + R_t^{H,s} \left(1 - \xi^{\text{ap}}\right). \tag{15}$$

Combining this equation with that describing the relative transmissibility ($R_t^{H,a} = \chi R_t^H$) gives an expression for the rescaled transmissibility of symptomatic infections:

$$R_t^{H,s} = \underbrace{\left(\frac{1 - \chi \xi^{\text{ap}}}{1 - \xi^{\text{ap}}}\right)}_{\chi^s} R_t^H. \tag{16}$$

To analyze the stability of this system, we need to write detailed equations for all the compartments, as schematized in 1. After linearizing the system in the shape $x' = Ax$, where $x = (T^s, T^a, H^s, H^a)$ and A is given by equation 17, we study its eigenvalues. Critical values of the reproduction number R_{crit}^H are presented in Supplementary Fig. 6.

$$A = \begin{pmatrix} \xi^s \nu R_t^H \Gamma \chi^s - \Gamma & \xi^s \nu R_t^H \Gamma \chi & (\lambda_r + \lambda_s)(1 + \xi^s \eta R_t^H \chi^s) & \xi^s R_t^H \eta \lambda_r \chi \\ \xi \nu R_t^H \chi^s \Gamma & \xi \nu R_t^H \Gamma \chi - \Gamma & (\lambda_r + \lambda_s) \eta R_t^H \xi \chi^s & \xi R_t^H \eta \lambda_r \chi + \lambda_r \\ \xi^s \epsilon R_t^H \Gamma \chi^s & \xi^s \epsilon R_t^H \Gamma \chi & -(\lambda_r + \lambda_s)(1 + \xi^s \eta R_t^H \chi^s) - \Gamma + \xi^s R_t^H \Gamma \chi^s & -\xi^s R_t^H \eta \lambda_r \chi + \xi^s R_t^H \Gamma \chi \\ \xi \epsilon R_t^H \Gamma \chi^s & \xi \epsilon R_t^H \Gamma \chi & -\xi \eta R_t^H (\lambda_r + \lambda_s) \chi^s + \xi R_t^H \Gamma \chi^s & -\lambda_r - \xi R_t^H \eta \lambda_r \chi + \xi R_t^H \Gamma \chi - \Gamma \end{pmatrix} \tag{17}$$

where $\xi^s = 1 - \xi$ and $\chi^s = (1 - \chi \xi)/\xi^s$.

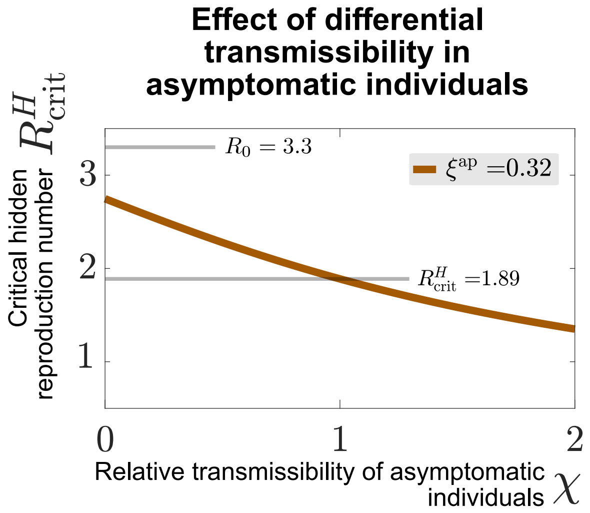

Supplementary Figure 6: **Effect of differential transmissibility of asymptomatic and symptomatic infections on the critical reproduction number.** This figure is similar to those in Supplementary Fig. 2A, but explores the impact of an additional parameter: the relative transmissibility factor χ accounts for the fact that asymptomatic individuals might be less ($\chi < 1$) or more ($\chi > 1$) infectious than symptomatic individuals. The solid brown curve shows the critical reproduction number computed from linear stability analysis (equation 17). Dashed lines show the default value for $\chi = 1$ and the basic reproduction number R_0, respectively.

Supplementary References

[1] Liu, Y., Gayle, A. A., Wilder-Smith, A. & Rocklöv, J. The reproductive number of COVID-19 is higher compared to SARS coronavirus. *Journal of travel medicine* **27**, taaa021 (2020).

[2] Alimohamadi, Y. *et al.* Estimate of the Basic Reproduction Number for COVID-19: A Systematic Review and Meta-analysis. *J Prev Med Public Health* **53**, 151–157 (2020).

[3] Barber, A. *et al.* The basic reproduction number of SARS-CoV-2: a scoping review of available evidence. Preprint at URL `https://doi.org/10.1101/2020.07.28.20163535` (2020).
