# Supplementary material for: The challenges of containing SARS-CoV-2 via test-trace-and-isolate
Source: Nat Commun. 2021 Jan 15;12:378. doi: 10.1038/s41467-020-20699-8 (PMC7810722; doi:10.1038/s41467-020-20699-8)
Supplement: Supplementary file 3 — Reporting Summary [file 41467_2020_20699_MOESM3_ESM.pdf]

nature research

Corresponding author(s): Viola Priesemann

Last updated by author(s): Nov 25, 2020

Reporting Summary

Nature Research wishes to improve the reproducibility of the work that we publish. This form provides structure for consistency and transparency in reporting. For further information on Nature Research policies, see our Editorial Policies and the Editorial Policy Checklist.

Statistics

For all statistical analyses, confirm that the following items are present in the figure legend, table legend, main text, or Methods section.

n/a	Confirmed	
☐	☒	The exact sample size (*n*) for each experimental group/condition, given as a discrete number and unit of measurement
☒	☐	A statement on whether measurements were taken from distinct samples or whether the same sample was measured repeatedly
☒	☐	The statistical test(s) used AND whether they are one- or two-sided<br>*Only common tests should be described solely by name; describe more complex techniques in the Methods section.*
☒	☐	A description of all covariates tested
☒	☐	A description of any assumptions or corrections, such as tests of normality and adjustment for multiple comparisons
☐	☒	A full description of the statistical parameters including central tendency (e.g. means) or other basic estimates (e.g. regression coefficient) AND variation (e.g. standard deviation) or associated estimates of uncertainty (e.g. confidence intervals)
☒	☐	For null hypothesis testing, the test statistic (e.g. *F*, *t*, *r*) with confidence intervals, effect sizes, degrees of freedom and *P* value noted<br>*Give P values as exact values whenever suitable.*
☒	☐	For Bayesian analysis, information on the choice of priors and Markov chain Monte Carlo settings
☒	☐	For hierarchical and complex designs, identification of the appropriate level for tests and full reporting of outcomes
☒	☐	Estimates of effect sizes (e.g. Cohen's *d*, Pearson's *r*), indicating how they were calculated

Our web collection on statistics for biologists contains articles on many of the points above.

Software and code

Policy information about availability of computer code

Data collection	MATLAB 2020a (https://github.com/Priesemann-Group/covid19_tti and Supplementary Dataset 1) All data used in this study is generated in the plotting routines, available in the GitHub repository. Alternatively, an interactive platform for simulating scenarios different from the herein presented is available on http://covid19-tti.ds.mpg.de, and users may download the data generated. DOI: 10.5281/zenodo.4290679
Data analysis	MATLAB 2020a (https://github.com/Priesemann-Group/covid19_tti). DOI: 10.5281/zenodo.4290679

For manuscripts utilizing custom algorithms or software that are central to the research but not yet described in published literature, software must be made available to editors and reviewers. We strongly encourage code deposition in a community repository (e.g. GitHub). See the Nature Research guidelines for submitting code & software for further information.

Data

Policy information about availability of data

All manuscripts must include a data availability statement. This statement should provide the following information, where applicable:

- Accession codes, unique identifiers, or web links for publicly available datasets
- A list of figures that have associated raw data
- A description of any restrictions on data availability

Data used in this study was obtained through numerical simulation, and it is available together with the code for solving our model's equations for default and user-customized parameters at https://github.com/Priesemann-Group/covid19_tti. Alternatively, an interactive platform for simulating scenarios different from the herein presented is available on http://covid19-tti.ds.mpg.de, and users may download the data generated.

Field-specific reporting

Please select the one below that is the best fit for your research. If you are not sure, read the appropriate sections before making your selection.

- [ ] Life sciences
- [x] Behavioural & social sciences
- [ ] Ecological, evolutionary & environmental sciences

For a reference copy of the document with all sections, see nature.com/documents/nr-reporting-summary-flat.pdf

Behavioural & social sciences study design

All studies must disclose on these points even when the disclosure is negative.

Study description	Quantitative modelling study on the spreading dynamics of COVID-19.
Research sample	Numerical experiments using empirical epidemiological parameters tailored to the case example Germany, given the experience of the group working with local data.
Sampling strategy	No sample size was calculated for Supplementary Figure 2. As input distributions in the error propagation were well-known, and the resulting distribution was smooth, a sample size of 10^(5) seemed reasonable.
Data collection	Empirical epidemiological parameters were selected to resemble, through simulation, expected and observed trends in Germany. All data, besides those parameters that are explicitly referenced in the manuscript, was obtained through simulation.
Timing	n/a, as selected parameters do not have a temporal dependency and all data was obtained through simulation.
Data exclusions	No data were excluded from the analyses.
Non-participation	n/a, as our manuscript presents a simulation-based study.
Randomization	n/a, as our manuscript presents a simulation-based study, where the effect of parameters was linked deterministically with the outcome.

Reporting for specific materials, systems and methods

We require information from authors about some types of materials, experimental systems and methods used in many studies. Here, indicate whether each material, system or method listed is relevant to your study. If you are not sure if a list item applies to your research, read the appropriate section before selecting a response.

Materials & experimental systems

n/a	Involved in the study	
☒	☐	Antibodies
☒	☐	Eukaryotic cell lines
☒	☐	Palaeontology and archaeology
☒	☐	Animals and other organisms
☒	☐	Human research participants
☒	☐	Clinical data
☒	☐	Dual use research of concern

Methods

n/a	Involved in the study	
☒	☐	ChIP-seq
☒	☐	Flow cytometry
☒	☐	MRI-based neuroimaging
